# Emergence of Two different recombinant PRRSV strains with low neutralizing antibody susceptibility in China

**DOI:** 10.1038/s41598-019-39059-8

**Published:** 2019-02-21

**Authors:** Guangwei Han, Huiling Xu, Kexiong Wang, Fang He

**Affiliations:** 1 Institute of Preventive Veterinary Medicine, College of Animal Sciences of Zhejiang University, Hangzhou, China; 2 Zhejiang Zhengli Antoo Biotech. Co.,Ltd, Ningbo, China

## Abstract

PRRSV causes major economic loss in global swine industry. 41 of 131 (31.29%) tissue samples collected from pig farms in central and east China from 2016 to 2017 were confirmed as PRRSV positive in RT-PCR. Base on phylogenetic analysis for ORF5 and ORF6, 3 isolates closely related to QYYZ strain form a new subgroup IV, while 3 other ones were clustered into subgroup III, represented by NADC30. Numerous amino acid substitutions involved in viral neutralization susceptibility were identified in GP5 among these isolates. Two emerging PRRSV strains (ZJnb16-2, SDbz16-2) were successfully isolated and sequenced. ZJnb16-2 was identified as a recombinant virus between strain QYYZ and JXA1 while SDbz16-2 was an inter-subgenotype recombinant virus of strains NADC30 and JXA1. As shown in the pathogenicity evaluation in piglets, ZJnb16-2 is highly pathogenic while SDbz16-2 is mild. Hyper-immune sera against major vaccine strains HUN4-F112 and JK-100 failed to neutralize either ZJnb16-2 or SDbz16-2. Only 0.8–2.0% of pig serum samples which were confirmed as PRRSV-positive with commercial ELISA kits presented neutralization reactivity against either ZJnb16-2 or SDbz16-2. The study confirmed that the viral genomic recombination contributes to the emergence of new pathogenic PRRSVs in China, which may escape from the protective immunity elicited by the conventional vaccines, highlighting the necessity in updates of vaccine strains and the need for a universal vaccine against PRRSV.

## Introduction

Porcine reproductive and respiratory syndrome (PRRS) is caused by the PRRS virus (PRRSV) leading to severe economic loss in the swine industry worldwide. PRRSV is an enveloped single stranded positive-sense RNA virus of the Arteriviridae family within the Arterivirus genus^1,2^. The genome is approximately 15 kb in length and possesses 9 open reading frames (ORFs) as well as 2 un-translated regions located in the 5′ and 3′ ends of the genome^3,4^. ORF1a and ORF1b encode polyproteins pp1a and pp1b which can be processed into 17 nonstructural proteins (NSP1α, NSP1β, NSP2, NSP2TN, NSP2NF, NSP3-14) at last involved in viral replication and transcription^5,6^. ORF2a, ORF2b, and ORFs 3–7 encode the viral structural proteins, GP2a, GP2b, GP3, GP4, GP5, GP5a, M, and N, respectively^7–9^.

PRRSV is classified into two genotypes, European (type 1) and North American (type 2), with Lelystad and VR-2332 as representative strains, respectively^10^. Although the two genotypes cause similar clinical symptoms, PRRSV isolates display only approximately 60% nucleotide identity to each other. In China, PRRSV was first isolated in 1995 which was clustered as genotype type 2. In 2006, HP-PRRSV (highly pathogenic PRRSV) was reported as the main cause of porcine high-fever disease associated with high mortality in China and quickly spread throughout the country^11,12^. In 2010, the lineage 3 PRRSV emerged in Southern China represented by QYYZ^13,14^. From 2013, diverse NADC30-like PRRSVs have been reported in several provinces of China resulted from the recombination between the NADC30 strain and the classic PRRSV, HP-PRRSV strains or both in the field^15,16^. Obviously, the diversity of type 2 PRRSV in China is currently increasing related to recombination events among field strains or between field and MLV vaccine strains.

To understand the molecular epidemiology for PRRSV in Central and eastern China from 2016 to 2017, a total of 131 tissue samples were collected from piglets in provinces Zhejiang, Shandong, Jiangsu and Henan. ORF5, M and Nsp2 genes of 41 PRRSV isolates were sequenced for amino acid variation and phylogenetic analysis. Furthermore, two novel isolates named ZJnb16-2, SDbz16-2 were completely sequenced showing different recombination events between JXA1 and QYYZ, NADC30 strains. In addition, the pathogenicity of the two strains was evaluated in pigs. The reactivity of ZJnb16-2, SDbz16-2 with neutralizing antibody against HP-PRRSV was also assessed.

## Results

### Phylogenetic analysis of novel strains and Amino acid analysis of GP5, M

**Figure 1:**
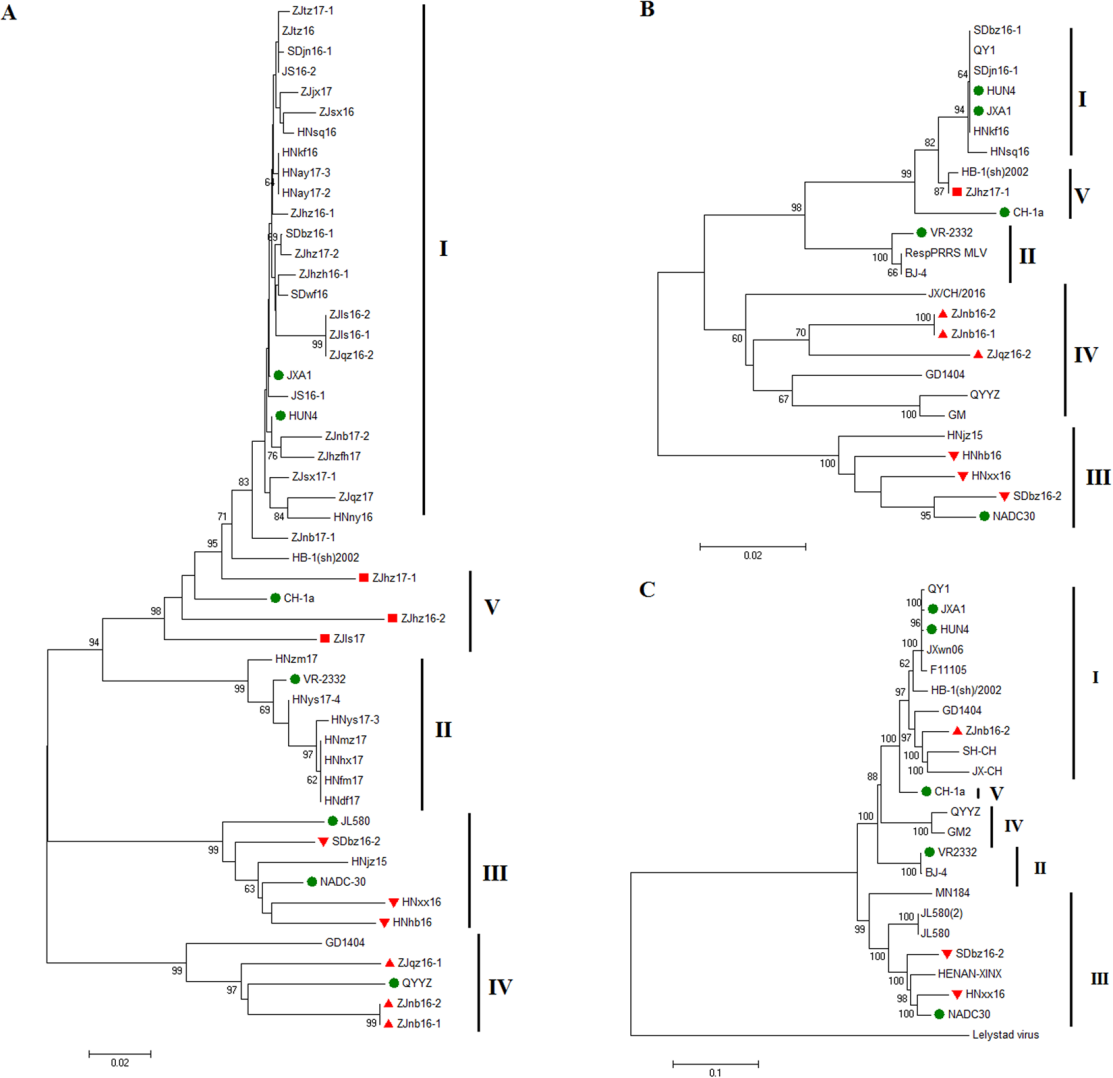
Phylogenetic tree based on ORF5 genes (**A**), ORF6 genes (**B**), complete genome of ZJnb16-2 and SDbz16-2 (**C**) and reference viruses. Trees were constructed using the neighbor-joining method with the maximum composite likelihood method (1000 replicates for bootstrapping). The reliability of the tree was assessed by a bootstrap analysis with 100 replications. Reference strains were labeled with a green solid circle. Isolated strains were labeled with red triangle and square.

From 131 samples collected from provinces Zhejiang, Jiangsu, Shandong, Henan of China, 41 samples (31.29%) were positive for PRRSV detected by RT-PCR. Totally 41 ORF5, 10 ORF6 and 13 NSP2 genes were amplified and sequenced for further analysis. Strain names, areas and accession numbers are summarized in Table 1. Phylogenetic analysis was performed on GP5 of 41 PRRSV strains and 17 reference strains. As shown in Fig. 1A, all the isolates from this study can be clustered into five subgroups. The majority (25/41) were grouped into the JXA1-like sub-genotype with multiple branches, and 7 isolates were grouped in the VR-2332-like sub-genotype. 3 isolates from Zhejiang province formed a new cluster subgroup IV represented by QYYZ. 3 isolates were clustered into subgroup III represented by NADC30. The result of phylogenetic analysis of M protein was consistent with the one of GP5 (Fig. 1B).

**Table 1:** Geographic origin and accession number of clinical samples in this study.

NO	Isolate	area	year	Accession no		
				ORF5	ORF6	NSP2
1	ZJqz16-1	Zhejiang	2016	MH174983	/	MH588703
2	ZJqz16-2	Zhejiang	2016	MH174984	MH588691	MH588702
3	ZJnb16-1	Zhejiang	2016	MH174985	MH588692	MH588704
4	ZJnb16-2	Zhejiang	2016	MH174986	MH588693	MH588705
5	ZJls16-1	Zhejiang	2016	MH174987	/	MH588706
6	ZJls16-2	Zhejiang	2016	MH174988	/	MH588707
7	ZJsx16	Zhejiang	2016	MH174989	/	/
8	ZJtz16	Zhejiang	2016	MH174990	/	MH588708
9	ZJhz16-1	Zhejiang	2016	MH174991	/	/
10	ZJhz16-2	Zhejiang	2016	MH174992	/	/
11	ZJhzh16-1	Zhejiang	2016	MH174993	/	/
12	HNhb16	Henan	2016	MH174994	MH588696	/
13	HNkf16	Henan	2016	MH174995	MH588697	/
14	HNny16	Henan	2016	MH174996		/
15	HNsq16	Henan	2016	MH174997	MH588694	/
16	HNxx16	Henan	2016	MH174998	MH588695	MH588709
17	SDwf16	Shandong	2016	MH175004		/
18	SDjn16-1	Shandong	2016	MH175003	MH588690	/
19	SDbz16-1	Shandong	2016	MH175002	MH588689	/
20	SDbz16-2	Shandong	2016	MH175001	MH588710	MH588710
21	JS16-2	Jiangsu	2016	MH174999	/	/
22	JS16-1	Jiangsu	2016	MH175000	/	/
23	ZJnb17-1	Zhejiang	2017	MH175005	/	MH588701
24	ZJhz17-1	Zhejiang	2017	MH175006	/	MH588699
25	ZJhz17-2	Zhejiang	2017	MH175007	/	MH588698
26	ZJnb17-2	Zhejiang	2017	MH175008	/	/
27	ZJsx17-1	Zhejiang	2017	MH175009	/	/
28	ZJhz17	Zhejiang	2017	MH175011	/	MH588700
29	ZJjx17	Zhejiang	2017	MH175012	/	/
30	ZJls17	Zhejiang	2017	MH175013	/	/
31	ZJqz17	Zhejiang	2017	MH175014	/	/
32	ZJtz17	Zhejiang	2017	MH175015	/	/
33	HNay17-2	Henan	2017	MH175016	/	/
34	HNay17-3	Henan	2017	MH175017	/	/
35	HNys17-3	Henan	2017	MH175018	/	/
36	HNys17-4	Henan	2017	MH175019	/	/
37	HNdf17	Henan	2017	MH175020	/	/
38	HNfm17	Henan	2017	MH175021	/	/
39	HNhx17	Henan	2017	MH175022	/	/
40	HNmz17	Henan	2017	MH175023	/	/
41	HNzm17	Henan	2017	MH175024	/	/

Multiple alignments of GP5 amino acid sequences indicated that all 41 ORFs encode 200 amino acid residues with extensive substitutions. The primary neutralizing epitope (PNE) at aa37–44 of GP5 is important in inducing neutralizing antibody. As shown in Table 2, all the isolates in subgroup IV had two H38Y and L39S mutants as compared with VR2332. Mutations V102H, V102C, V102Y were observed among subgroup IV isolates. Isolates in subgenotype III have substitution G to E, K or R in aa104 as compared to VR2332 (Supplementary Fig. S1). These substitutions may greatly affect the antigenicity of GP5 protein and the cross reactivity in neutralization. In this study, mutation H10N was observed among subgenotype I, III and II, IV, V in M protein (Table 2).

**Table 2:** Distribution of neutralization epitope associated residues within GP5 and M of isolated strains.

Subgroup	No. of isolates with indicated neutralization epitope associated residues/total no. of isolates (GP5 aa 37-45, M aa9-10)						
	H^38^	Y^38^	L^39^	I^39^	S^39^	N^10^	H^10^
I	26/26	0/26	0/26	26/26	0/26	26/26	0/26
II	7/7	0/7	7/7	0/7	0/7	0/7	7/7
III	3/3	0/3	0/3	3/3	0/3	0/3	3/3
IV	0/3	3/3	0/3	0/3	3/3	0/3	3/3
V	2/2	0/2	0/2	2/2	0/2	0/2	2/2
Total no.	38/41	3/41	7/41	31/41	3/41	26/41	25/41

The number of potential N-glycosylation sites in different subgroup was summarized in Table 3. All the isolates of different subgenotypes have two conserved N-glycosylation sites at positions 44 and 51. There are fewer glycosylation sites in subgenotype IV than other subgenotypes. N33 is rarely found in GP5 of subgenotype IV isolates. Most isolates from subgenotype II, III, V have 4 potential N-glycosylation sites except for two strains.

**Table 3:** The numbers of potential N-linked glycosylation sites in GP5 proteins of different PRRSV isolates.

Subgroup	No. of isolates with the indicated no. of potential N-glycosylation sites/total no. of isolates		
	3	4	5
I	0/26	1/26	25/26
II	0/7	7/7	0/7
III	0/3	3/3	0/3
IV	3/3	0/3	0/3
V	1/2	1/2	0/2
Total no.	4/41	12/41	25/41

### Analysis of full-length genomic sequences of isolates ZJnb16-2 and SDbz16-2

Genomic sequences of ZJnb16-2 (GenBank accession number: MH236416), SDbz16-2 (GenBank accession number: MH588710) were 15,307 and 15,020 nucleotides in length respectively, excluding the poly (A) tails. Phylogenetic analysis of ZJnb16-2 and SDbz16-2 was constructed based on the complete nucleotide sequences with 17 reference PRRSV strains of type 2 available in GenBank (Supplementary Table S2). All PRRSV strains were divided into five subgroups (I-V). ZJnb16-2 is closely related with JXA1-like sub-genotype and SDbz16-2 is from NADC30-like sub-genotype (Fig. 1C). Multiple sequence alignment of NSP2 indicated that ZJnb16-2 had 30 discontinuous deletions which are similar to HP-PRRSV. 4 unique continuous deletions between aa 797–800 was observed in NSP2 coding area of ZJnb16-2 (Supplementary Fig. S2). Besides, the deletion of discontinuous 131 amino acids was observed in SDbz16-2, which was found in previous NADC30-like PRRSVs (Supplementary Fig. S2). ZJnb16-2 presented relatively low identities in ORF2-7 (83.9–92.8%) with JXA1 at nucleotide sequences (Table 4). SDbz16-2 has also lower homology (78.7–92%) with JXA1 at the amino acid level of structural protein 2–5 (Table 4).

**Table 4:** Nucleotide and amino acid identities of different regions of ZJnb16-2 and SDbz16-2 compared with other PRRSV reference strains.

Regions	JXA1		VR2332		QYYZ		NADC30		CH-1a	
	ZJnb16-2	SDbz16-2	ZJnb16-2	SDbz16-2	ZJnb16-2	SDbz16-2	ZJnb16-2	SDbz16-2	ZJnb16-2	SDbz16-2
genome	93.9	82.6	87.4	83.1	85.9	79.3	81.9	94.6	90.8	82.7
5′UTR	98.4	89.5	93.6	91.6	94.2	88.5	92.6	95.3	96.3	91.1
ORF1a	96.1/95.4	78.3/77.8	86.4/86.1	78.4/78.2	82/82.4	72.8/72.9	80.7/82	92.9/93.7	92/90.9	78/77
ORF1b	94.7/97.9	87.1/95.1	89.6/95.7	87.8/94.9	89.2/96	85.8/94	86/91	96/97.9	92.7/98.5	87.6/94.9
ORF2a	90.3/89.1	85.6/85.9	91.2/90.6	87.7/89.5	93.1/93.4	86.1/89.5	87.2/88.7	95.7/94.5	91.2/89.5	86.5/86.7
ORF2b	92.8/91.8	87.4/87.7	92.8/91.8	89.2/86.3	95.5/94.5	90.5/87.7	91/91.8	94.6/93.2	93.2/91.8	87.4/86.3
ORF3	86.7/87.4	81.7/78.7	88.5/87.8	82.9/80.7	88.1/89.4	82/81.1	86.1/85.8	96.7/96.9	87.6/87.8	82.5/79.1
ORF4	86.4/90.4	84.9/86	88.8/90.4	87.3/86.5	88.3/93.3	84.9/85.4	87.3/88.2	97/96.6	88.1/92.7	86.4/87.1
ORF5	83.9/83.5	85.7/85.5	83.6/81.5	86.7/84.5	91.7/93	84.1/84	84.7/84.5	94.9/94	85.2/84	87.4/86.5
ORF6	90.7/95.4	88.4/92	92.6/95.4	89.5/93.1	92.4/96.6	89.9/93.1	90.7/93.1	97.9/97.7	89.7/95.4	88/92
ORF7	90.9/91.9	90.9/90.2	91.4/95.2	92.5/91.9	92.5/95.9	87.1/90.2	90.1/91.9	96/95.1	89.8/93.5	91.4/90.2
3′UTR	85.9	88.7	87.2	93.4	89.3	89.4	83.9	98.7	85.2	88.7

### Recombination analysis of ZJnb16-2 and SDbz16-2

**Figure 2:**
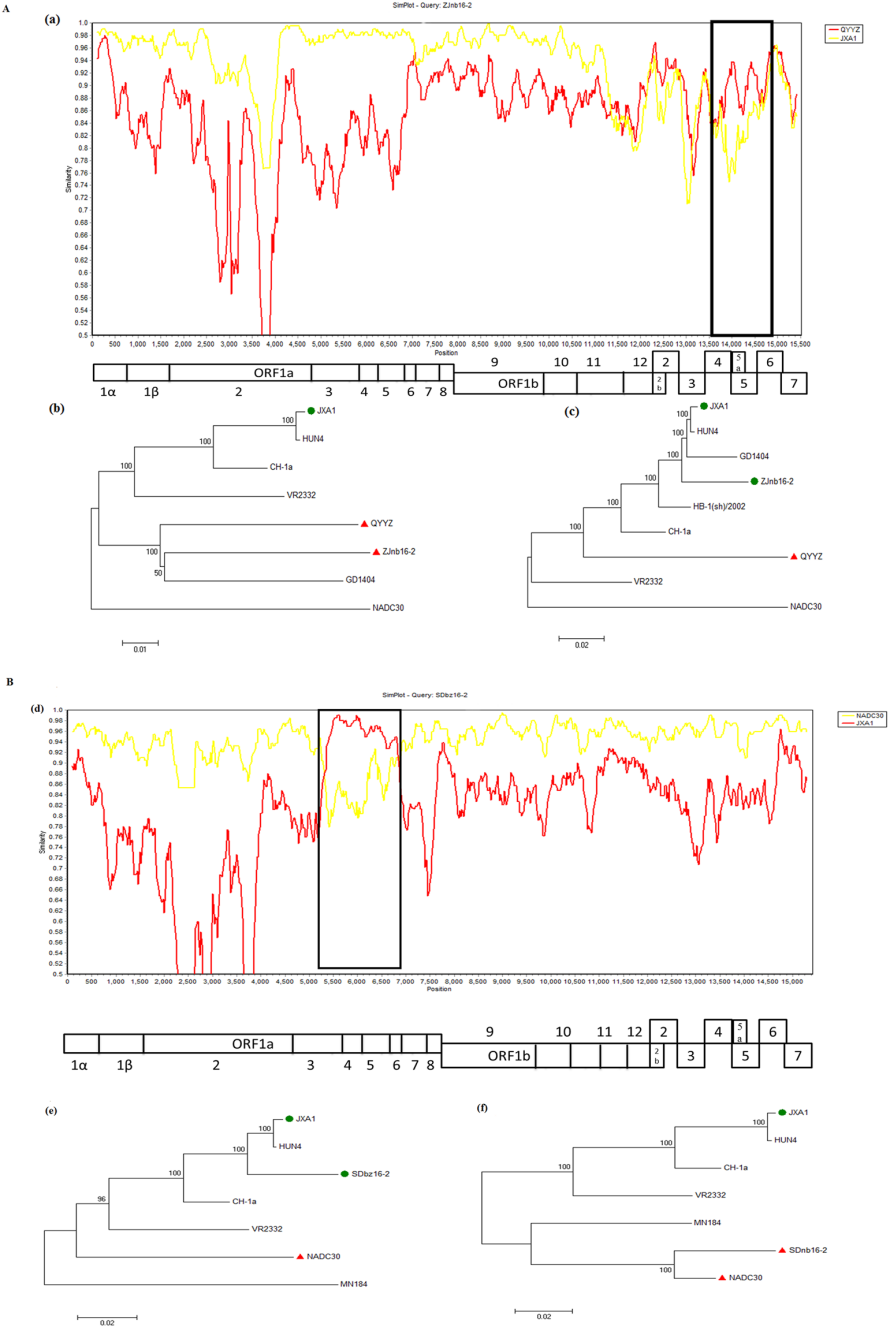
Recombination analysis of strain ZJnb16-2 (**A**), SDbz16-2 (**B**). Recombination analysis was calculated by Simplot 3.5.1 software. Complete genome of ZJnb16-2 and SDbz16-2 were chosen as the query sequence. Sequence included in the black box was the minor parental region while the rest was the major parental regions (a,d). A complete genome structure was shown under the similarity plot with reference to JXA1, of which the positions of the major ORFs and *Nsp*s were indicated. Phylogenies of the parental regions for ZJnb16-2(b,c), SDbz16-2 (e,f) were shown below the genome structure.

Similarity comparisons were performed using a sliding window of 500 bp along the genome alignment (20 bp step size) to analyze the recombination of ZJnb16-2, SDbz16-2. One breakpoint located in ORF2-ORF7 (nt11189-nt15509) of ZJnb16-2 genome, which separated the ZJnb16-2 genome into two regions, ORF2-ORF7 segment is similar to the lineage 3 represented by QYYZ and the remaining segments are closely related to the Chinese HP-PRRSV strains JXA1 (Fig. 2A). These data suggested that strain ZJnb16-2 is the product of a recombination event between strains JXA1 and QYYZ.

Meanwhile, one breakpoint was identified in the genome of SDbz16-2 located in nt5292 and nt6854 of NSP4-NSP5 coding region (Fig. 2B). SDbz16-2 genome was divided into three regions. The intermediate segment is similar to HP-PRRSV strains represented by JXA1 and the remaining segments are closely related to the NADC30. These results indicate that the PRRSV isolate SDbz16-2 is a recombinant virus between NADC30-like and JXA1-like PRRSV recently prevailing in China. The recombination events were confirmed by RDP4 software using RDP, MaxChi, 3Seq, GENECONV, Bootsacan method.

### Pathogenicity analysis of ZJnb16-2 and SDbz16-2

**Figure 3:**
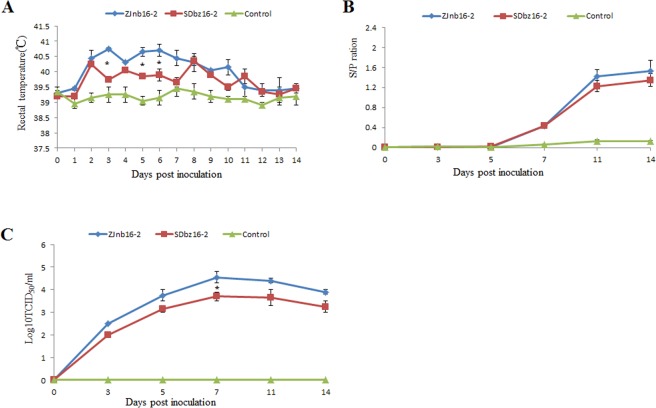
Pathogenicity study in pigs. (**A**) Rectal temperatures of piglets inoculated with ZJnb16-2, SDbz16-2, and 1640. Mean ± SD (error bars) temperatures. (**B**) PRRSV N protein antibody response of piglets was measured by ELISA. Samples were confirmed positive for antibody to PRRSV if the S/P ratio ≥0.4. The data were expressed as the mean ± S.D. of the numbers of pigs alive at the time of sample collection. (**C**) Virus titer from sera samples at each collection time point.

All inoculated piglets were monitored until 14 days post inoculation. All piglets in ZJnb16-2 group developed severe clinical symptoms including depression, anorexia, respiratory distress and shivering. In contrast, SDbz16-2-infected piglets showed moderate clinical signs including depression, anorexia symptoms only. The pigs infected with ZJnb16-2 isolates had higher rectal temperatures for 6 days than the SDbz16-2 group (Fig. 3A). No obvious clinical signs were observed in the control group during the entire experiment period. Specific antibodies against N protein of PRRSV in sera of all the inoculated piglets were detected in 7 days post infection (Fig. 3B). Antibody level of N protein in ZJnb16-2 group is higher than SDbz16-2 group in 11 and 14 days post infection. Virus titers in sera were calculated using the Reed–Muench method and results indicated that virus titers peaked at 7 days post infection in the two groups (Fig. 3C). Average virus titer in sera of ZJnb16-2 group was higher than SDbz16-2 group during the whole study (Fig. 3C).

**Figure 4:**
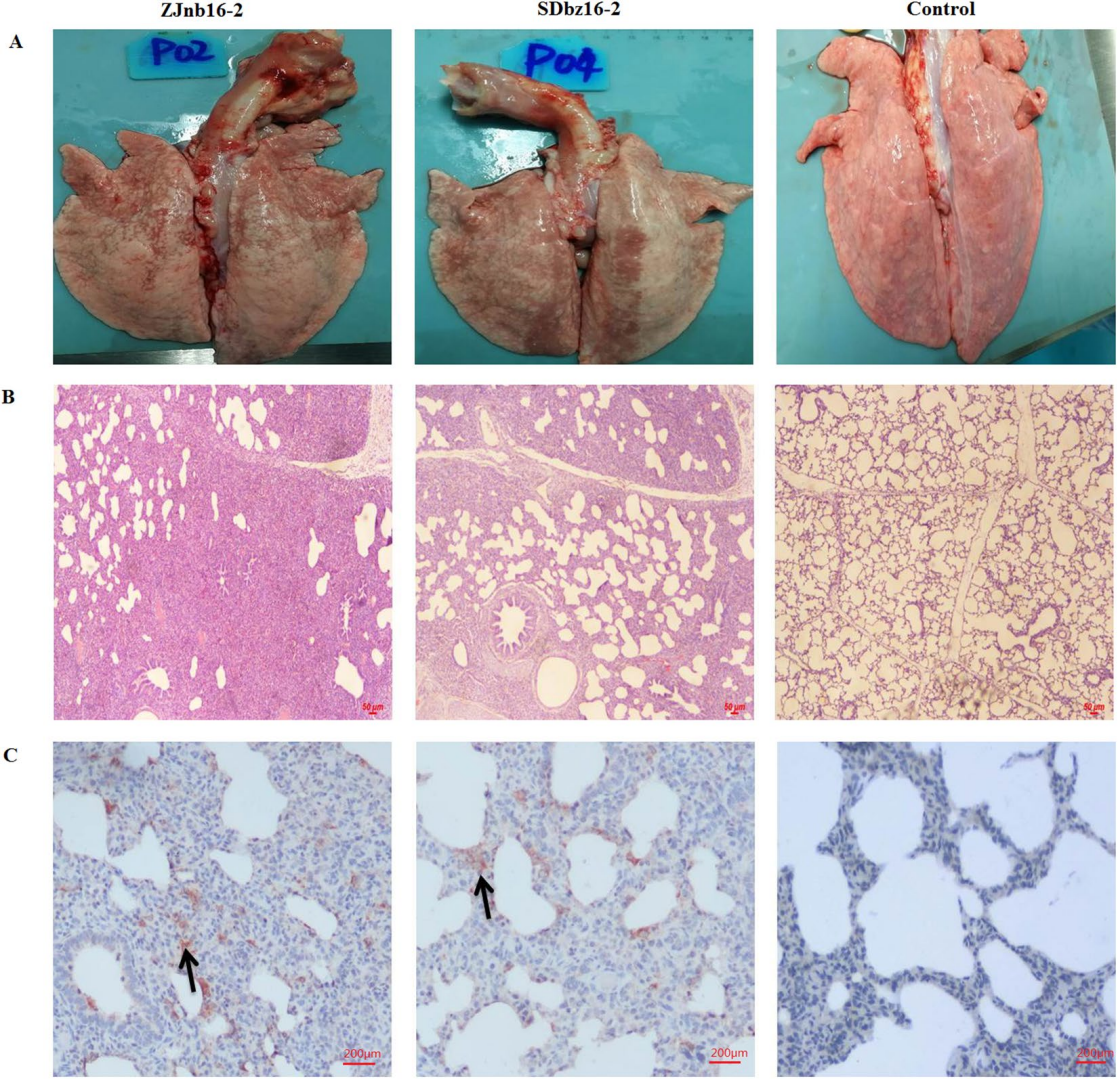
Lesions in infected lung tissue. (**A**) Gross lesions in the lung in each group. (**B**) Microscopic lesions in the lung of each experimental group. Lung tissues were stained with hematoxylin and eosin. (**C**) Lung tissues were examined by immunohistochemistry using monoclonal antibodies 9D9 against N protein of PRRSV.

All surviving pigs were euthanized and necropsied 14 days post inoculation. A fraction of lung consolidation was observed in both ZJnb16-2 and SDbz16-2 groups but not in the uninfected group (Fig. 4A). Histopathological examination indicated that interstitial pneumonia with infiltration of numerous inflammatory cells was observed in both ZJnb16-2 and SDbz16-2 group (Fig. 4B). ZJnb16-2 inoculated piglets presented moderate diffuse lung lesions. Intact lung structure was hardly observed in the ZJnb16-2 group as compare to the control group while SDbz16-2 infected group displayed scattered lung lesions only. PRRSV antigens in lungs were examined by immunohistochemistry with 9D9. As shown in Fig. 4C, PRRSV positive signals were observed in both infection groups. No PRRSV-specific signals were detected in lungs of control piglets.

### Virus cross-neutralization assay of ZJnb16-2 and SDbz16-2

**Figure 5:**
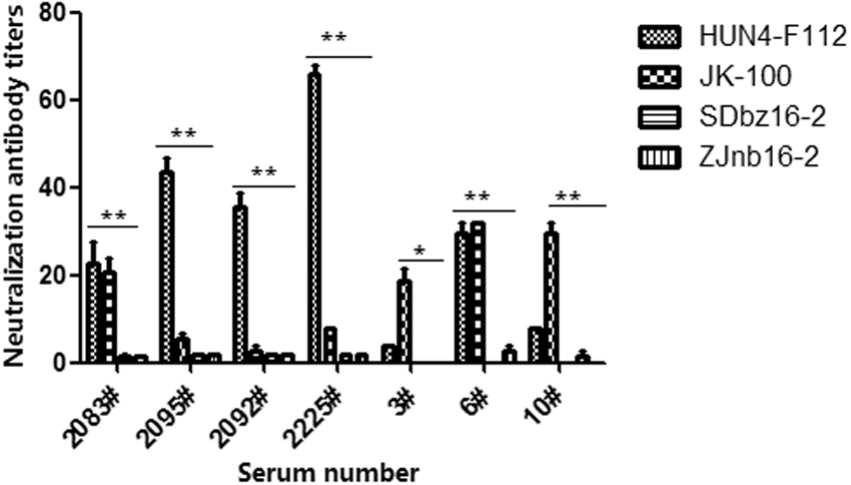
Cross neutralization assays with hyper-immune sera of conventional vaccine strains against ZJnb16-2 and SDbz16-2. The data are represented as the means ± S.D. of three independent experiments, and significant differences are shown (*P < 0.05 and ***P < 0.001) by using one-way analysis of variance.

Commercial PRRSV vaccine strains currently used in China were listed in Table 5. HUN4-F112 and JK100 (Accession number AF332733) were representative vaccine strains respectively for the classical and highly pathogenic PRRSV. Hyper-immune sera generated from HUN4-F112 and JK-100 were used for viral cross-neutralization against ZJnb16-2 and SDbz16-2. Neutralization titers of sera (2083#, 2095#, 2092# and 2225#) against HUN4-F112 ranged from 1:22 to 1:64, while JK-100 sera (1#, 6#, 10#) presented neutralizing titers from 1:18 to 1:32. However, all the hyper-immune sera fail to neutralize either ZJnb16-2 or SDbz16-2 (Fig. 5).

**Table 5:** Commercial PRRSV vaccines currently used in China.

Type of Vaccine	Vaccine strain	Subgroup
Inactivated vaccine	CH-1a	V
Attenuated vaccine (derived from Classical PRRSV)	ATCC VR-2332/JK100	II
	R98	II
	CH-1R	V
Attenuated vaccine (derived from Highly pathogenic PRRSV)	GDr180	I
	HuN4-F112	I
	TJM-F92	I
	JXA1-R	I

346 of 392 serum samples collected from Chinese local pig farms in 2016–2017 were confirmed as PRRSV-positive with a commercial PRRSV ELISA kit. Further, neutralization susceptibility of both ZJnb16-2 and SDbz16-2 were evaluated with the 346 PRRSV samples. As shown in Table 6, in different regions of China, more than 98% PRRSV-positive sera fail to neutralize the two new strains ZJnb16-2 and SDbz16-2 confirming that the neutralization evasion occurs to these emerging pathogenic PRRSVs.

**Table 6:** Cross-neutralization reactivity of clinical pig serum samples against ZJnb16-2 and SDbz16-2.

Areas	PRRSV-positive serum samples /Total serum samples	Positive neutralization against ZJnb16-2/PRRSV-positive serum samples	Positive neutralization against SDbz 16-2/PRRSV-positive serum samples
Shanxi	30/30 (100%)	0/30 (0%)	0/30 (0%)
Hebei	29/29 (100%)	0/29 (0%)	1/29 (3.4%)
Shandong	30/32 (93.7%)	0/30 (0%)	1/30 (3.3%)
Yunnan	12/12 (100%)	0/12 (0%)	0/12 (0%)
Hubei	15/17 (88.2%)	0/15 (0%)	0/15 (0%)
Jilin	4/12 (33.3%)	0/4 (0%)	0/4 (0%)
Hunan	26/30 (86.7%)	0/26 (0%)	0/26 (0%)
Fujian	25/32 (90%)	0/25 (0%)	1/25 (4%)
Sichuan	34/45 (75.6%)	2/34 (5.9%)	0/34 (0%)
Jiangsu	37/37 (100%)	0/37 (0%)	2/37 (5.4%)
Zhejiang	48/51 (94.1%)	1/48 (2.1%)	0/48 (0%)
Hainan	35/36 (97.2%)	0/35 (0%)	2/35 (5.7%)
Gansu	13/17 (76.4%)	0/13(0%)	0/13 (0%)
Jilin	8/12 (66.7%)	0/8 (0%)	0/8 (0%)
Total	346/392 (88.2%)	3/346 (0.8%)	7/346 (2.0%)

## Discussion

PRRS continues to be a severe threat to the swine industry worldwide. PRRSV was first isolated in 1996 and has spread widely in China. Nowadays, epidemic PRRSV type 2 strains in China can be divided into lineages 1, 3, 5.1 and 8.7 represented by NADC30-like, QYYZ-like, VR2332-like and JXA1-like, respectively^15^. From 2006, HP-PRRSV strain with the discontinuous 30aa deletion in NSP2 caused immense economic losses in China which may originate from CH-1a-like PRRSV^17^. In 2010, lineage 3 PRRSV was reported in China represented by QYYZ and GM2^13,14^. In 2013, NADC30-like PRRSV strains emerged in China^15,16,18^. Considering the high genetic and antigenic diversity of PRRSV isolates, it is necessary to continuously monitor emerging epidemic strains. In the present study, 13 *nsp2*, 10 ORF6 and 41 ORF5 genes of PRRSV isolates were sequenced originating from different swine farms in central and east China from 2016 to 2017. Phylogenetic analysis of GP5, M indicted that all the isolates from this study can be clustered into five subgroups. Most of isolates (25/41) were grouped into the JXA1-like subgroup and 4 isolates belonged to CH-1a-like subgroup. 3 isolates from Zhejiang province formed a new subgroup IV represented by QYYZ. Besides, NADC30-like isolates can be detected in Shandong and Henan province. The diversity of type 2 PRRSV in China is increasing and HP-PRRSVs are still the dominant epidemic strains consistent with the previous study^19^.

PRRSV is characterized by genetically extensive variation containing the mutation and recombination between different isolates. Recombination is considered to be the most important mechanism in the evolutionary history of PRRSVs. Two QYYZ-like recombination strains (GD1404, GDsg) between QYYZ and JXA1 were isolated in China in 2014 and 2015 respectively^19,20^. Many recombinant strains with different recombination sites between NADC30-like and Chinese HP-PRRSVs, VR-2332, or CH-1R have been reported in China^21,22^. Most of the recombination sites in these isolates are located in the coding regions of non-structure proteins^23^. In the present study, recombination analysis showed that strain ZJnb16-2 is the product of a recombination event between strains JXA1 and QYYZ. Recombination region of ZJnb16-2 is located in the structural protein coding area (ORF2-7), which is different from a QYYZ-like strain GDsg. SDbz16-2 is a recombinant virus emerging from the recombination event between NADC30-like and the Chinese HP-PRRSV-JXA1 and the recombination region is located in NSP4-5. Results in phylogenetic analysis of the whole genome indicated that ZJnb16-2 is closely related to JXA1 which is with the similar pathogenicity to HP-PRRSVs. However, the pathogenicity study indicated all piglets infected with ZJnb16-2 survived, while usually infection with other HP-PRRSV strain leads to lethal cases in pigs^24,25^. The attenuation in pathogenicity of ZJnb16-2 may be caused by the significant changes in sequences of structural proteins involved in attachment to alveolar macrophages for PRRSV^26^. Further study should be performed to confirm the changes in pathogenicity using reverse genetics technique. Pathogenicity of SDbz16-2 was also assessed in this study. Similar to most of NADC30-like isolates, SDbz16-2 displayed moderate virulence in this study. Different recombination pattern may affect the virulence of epidemic PRRSV isolates. The NADC-30 like isolate JL580 was more pathogenic than another NADC-30 like strain HNjz15, which were caused by the different recombination patterns^19,27^. According to the infection study, ZJnb16-2 caused severer clinical symptoms, lung lesions, viremia and higher rectal temperature in piglets than the SDbz16-2 group. The pathogenicity of QYYZ-like isolate ZJnb16-2 for piglets was obviously higher than the NADC-30 like isolate SDbz16-2, which may be related to the different parents and recombination regions.

GP5 is one of the most important structural proteins in PRRSV and plays a critical role in the regulation of immune response. aa37–44 of GP5 is an important neutralizing epitope which determines the neutralization reactivity in sera^28^. Besides, aa102 and aa104 of GP5 play a role in viral neutralization susceptibility^29^. In our study, all the isolates in subgroup IV had two mutations H38Y and L39S as compared to the VR2332 strain. Mutations V102H, V102C and V102Y were also observed among subgroup IV isolates as compared to VR2332. Glycosylation of GP5 is involved in viral infection, antigen characteristics and neutralization susceptibility of the virus^30^. There are fewer NGS numbers in subgroup IV. A Tyr-10 deletion in M protein allows virus evasion from broad neutralization^31^. In this study, mutation H10N was observed among subgenotype I and III as compared to II, IV and V. These variations may contribute to virus evasion from neutralization. Based on results of multiple sequence alignment, significant variation was identified in GP5 protein sequence of ZJnb16-2 and SDbz16-2. ZJnb16-2 shares 93% identity with QYYZ while SDbz16-2 shares 95.1% identity with NADC30 at amino acid sequence level of GP5. ZJnb16-2 and SDbz16-2 shares only 83.5% or 85.5% identity with JXA1 at amino acid sequence level of GP5, respectively. The variation in GP5 suggested the low neutralizing reactivity of ZJnb16-2 and SDbz16-2 to the conventional vaccine immunity.

Currently, commercial MLV vaccines are widely used for the prevention and the control of PRRS in China, including VR2332, R98, TJMF92, CH-1R, HUN4-F112 and JXA1-R. Previous studies indicated that the HP-PRRSV and C-PRRSV vaccine could not provide complete protection against NADC30-Like strains and cross humoral immunity to epidemic isolates could not be detected among immunized piglets^32^. In this study, HUN4-F112 is an attenuated vaccine strain sharing 99.6% identity with JXA1, while JK-100 is a naturally attenuated isolates sharing 99.8% identity with VR-2332 in nucleotide sequence. Efficient elicitation of neutralization antibody was detected in pigs immunized with HUN4-F112 and JK-100. However, hyper-immune sera against HUN4-F112 and JK-100 fail to neutralize isolated either ZJnb16-2 (QYYZ-like) or SDbz16-2 (NACDC-like), which may be caused by the high variation in GP5. Further, results of neutralization tests with 346 PRRSV-positive sera indicated that more than 99.8% of these PRRSV sera from China farms failed to neutralize the two new strain ZJnb16-2 and SDbz16-2, indicating that the neutralization evasion occurs to these emerging pathogenic PRRSVs. This study confirmed the heterologous humoral immunity with the conventional vaccine strains is weak, if not null, against circulating isolates, including those new recombinant strains.

Due to the high genetic and antigenic diversity, current vaccines could not provide complete protection against heterologous PRRSV^33^. Additional problems of conventional PRRSV vaccines include viral persistence and possible reversion to virulence, which contributes to the increase in the rate of recombination^22,24^. Hence, the study here indicated a new subgroup represented by QYYZ emerged in Zhejiang province. Two PRRSV strains (ZJnb16-2 and SDbz16-2) with different recombination pattern were isolated and the pathogenicity in piglets is divergent. ZJnb16-2 has the 30aa discontinuous deletion as HP-PRRSV and a unique continuous deletion of 4 amino acids between aa797 and aa800 in the Nsp2 coding region as compared with VR2332. These findings highlighted the necessity to continuously monitor the emergence of new PRRSVs from the recombination between MLV and field virus in pig farms and the needs for new vaccine development which could provide cross protection against different PRRSVs. Broadly neutralizing antibodies are central to effective control of PRRSV, which could prevent piglets from further infection^34^. Previously, based on the sequence analysis of major PRRSV strains, DNA shuffling of the GP4 and M genes from different parental viruses broaden the cross-neutralizing antibody-inducing ability of the chimeric viruses against heterologous PRRSV strains^35^. Besides, together with route PRRSV surveillance in China, engineering in conserved cross-neutralization epitopes of GP2, GP5 and M to improve the cross immunogenicity will be the alternative strategy for effective universal vaccines against PRRSV.

## Material and Methods

### Sample RT-PCR

A total of 131 lung and lymph node samples were collected from Zhejiang, Jiangsu, Shandong, Henan province of China from 2016 to 2017. Piglets displayed significant clinical signs including fever, labored breathing, lethargy and anorexia. Total RNA was extracted from supernatant using TRIzol reagent (Vazyme, China) according to the manufacturer’s instructions. First-strand cDNA was constructed by HiScript reverse transcriptase (Vazyme, China) with 3ul RNA used as a template in the subsequent PCR. The primers ORF5-F (5′-CATTTCATGACACCTGAGACCAT-3′) and ORF5-R (5′-GCATATATCATCACTGGCGTGTAGGT-3′), ORF6-F (5′-ATCGACCTCAAGAGAGTTGTGCTTGAT-3′), ORF6-R (5′-ACAGCTGATTGACTGGCTGGCCAT-3′) were used to amplify the whole ORF5 (776 bp) and ORF6 gene (649 bp). Partial Nsp2 gene was amplified with N-SP2-F (5′- CTCCAGATGACTGGGCTACTGACG-3′) and NSP2-R (5′- ATGATGGCTTGAGCTGAGTAG-3′). PCR products were purified with a Cycle Pure Kit (Omega, USA) and cloned into pEASY-Blunt Zero Vector (Transgen, China). At least three positive clones were sequenced using Sanger sequencing app-roach.

### Virus isolation and complete genome sequencing

Lung and lymph node samples were homogenized in 1640 medium (Gibco, Carlsbad, CA, USA) supplemented and subjected to three freeze-thaw cycles. After centrifugation, supernatant was passed through a 0.22-μm filter. Primary porcine alveolar macrophages (PAM) obtained from specific pathogen free piglets were used to isolate PRRSV pathogen from tissue samples. After inoculation, cells were maintained in Roswell Park Memorial Institute 1640 supplemented with 10% FBS and 1% antibiotic–antimycotic solution under a humidified 5% CO2 atmosphere at 37 °C. PRRSV infection was confirmed by CPE observation daily and indirect immunofluorescence assay (IFA) with PRRSV N protein specific antibody 9D9. After five more passages in PAMs, the collected viruses were designated ZJnb16-2 and SDbz16-2 for further complete genome sequencing.

Whole genome sequences of ZJnb16-2 and SDbz16-2 were amplified by 7 respective pairs of primers (Supplementary Table S1) in RT-PCR producing 7 overlapped fragments. PCR products were purified with a Cycle Pure Kit (Omega, USA) and cloned into pEASY-Blunt Zero Vector. At least three positive clones were sequenced using Sanger sequencing approach. Sequences of fragments from ZJnb16-2, SDbz16-2 were assembled into full-length consecutive genome by Vector NT1 software.

### Comparative genome and Phylogenetic analysis

Multiple sequence comparisons were performed using MegaAlign program in DNAstar 7.0 software and BioEdit program (v7.0.5, Borland, Scotts Valley, CA, USA) to determine sequence homology and amino acid mutation.

Phylogenetic trees based on full-length genome, ORF5 and ORF6 nucleotide sequences were generated with MEGA 7.0 software using the distance-based neighbor-joining method^36^. Bootstrap values were evaluated from 1000 replicates. Selected PRRSV reference strains and the detailed information are shown in Supplementary Table S2.

### Whole-Genome recombination analysis

Recombination events were detected by SimPlot software v.3.5.1^37^ and boot scanning analysis was performed with a window size of 200 bp and a step size of 20 bp. A recombination detection program (RDP v.4.24) was used to confirm the possible recombination event and the potential parental PRRSV lineages^38^. To verify the recombination, re-sequencing around the regions of the putative recombination breakpoints was performed.

### Pathogenicity study

All methods were performed in accordance with relevant guidelines and regulations. 9 6-week-old healthy landrace piglets were selected which are free of PRRSV, porcine circovirus type 2 (PCV2), classical swine fever virus (CSFV) and pseudorabies virus (PRV) before the experiment and randomly divided into three groups (n = 3). Each piglet in ZJnb16-2 or SDbz16-2 inoculated group was intranasally administered with 2 ml of virus containing 2.5 × 10^6^ TCID50. Control group was inoculated with PAM culture supernatant.

After inoculation, the pigs were monitored daily for general health status and rectal temperature. Sera samples were collected on 0, 1, 3, 5, 7, 10, 14 days post inoculation. All serum samples were serial diluted to determine TCID50. Antibodies against PRRSV N protein in sera were detected using a commercial IDEXX Herdchek PRRS 2XR ELISA kit (Westbrook, ME, USA). All the animals were euthanized at 14 dpi. Lung samples with consolidation were collected and fixed in 4% paraformaldehyde for histopathology and immunohistochemistry (IHC) examination. Fixed tissues were stained with hematoxylin and eosin (H&E) for pathological changes or with N protein monoclonal antibody 9D9 for viral antigen.

### Virus cross-neutralization assay

Four 6-week-old PRRSV-free landrace piglets were intramuscularly administered with 1 ml of PRRSV (1 × 10^7^ TCID_50_/ml). Hyper-immune sera was collected in 1–3 months post vaccination and used for cross-neutralization assays as described previously^39^. Briefly, sera were diluted using a twofold serial dilution technique in RPMI-1640 medium. 50 μl diluted sera were incubated with 100 TCID_50_ of virus at 37 °C and 5% CO2 for 1 hour. Mixtures were transferred into PAM cells prepared in 96-well plates and incubated for 24 hours. The presence of virus-infected cells in each well was determined by IFA using monoclonal antibody against N protein (9D9) of PRRSV and FITC-conjugated goat anti-mouse IgG (KPL, USA). Neutralizing antibody (NA) titers against the different PRRSV isolates were calculated using the Reed–Muench method^40^.

392 clinical serum samples were collected from pigs immunized with PRRSV commercial vaccines from 14 provinces in China. N protein antibody level was detected using a commercial IDEXX Herdchek PRRS 2XR ELISA kit. Serum samples were confirmed as positive in neutralization against ZJnb16-2 or SDbz16-2 with a neutralization titer ≥1:8.

### Statistical analysis

All data were expressed as mean ± S.D. and analyzed using GraphPad Prism software (version 5.0). Differences among groups were considered statistically significant when probability (p) value is less than 0.05.

### Ethical approval and informed consent

Animal experiments were approved by the Animal Welfare and Ethics Committee at Laboratory animal center of Zhejiang University (approve number 12095). All experiments were performed in accordance with relevant guidelines and regulations.

## Supplementary information

Dataset 1
