# Supplementary material for: Emergence of Two different recombinant PRRSV strains with low neutralizing antibody susceptibility in China
Source: Sci Rep. 2019 Feb 21;9:2490. doi: 10.1038/s41598-019-39059-8 (PMC6385303; doi:10.1038/s41598-019-39059-8)
Supplement: Supplementary file 1 — Dataset 1 [file 41598_2019_39059_MOESM1_ESM.pdf]

Emergence of Two different recombinant PRRSV strains with low neutralizing antibody susceptibility in China

Guangwei Han[1], Huiling Xu[1], Kexiong Wang[2], Fang He[1*]

[1] Institute of Preventive Veterinary Medicine, College of Animal Sciences of Zhejiang University, Hangzhou, China.

[2] Zhejiang Zhengli Antoo Biotech. Co.,Ltd. Ningbo, China.

Correspondence

*Fang He, Institute of Preventive Veterinary Medicine, College of Animal Sciences of Zhejiang University, 866 Yuhangtang road, Hangzhou, 310058, China

Email: hefangzj@zju.edu.cn

Supplementary Table S1

List of primers for ZJnb16-2(prime 1-7) and SDbz16-2(prime 8-16) used in this study

Primer	sequence	Nucleotides	Product size
1	F:ATGACGTATAGGTGTTGGCTCTATGCC	1-2398	2398bp
	R:GAATTTAACCTCTCACGGTGATGAACCTCGT		
2	F:CTACTCCGGAAGAGGTCGCGGCAAAGAT	2077-4838	2762bp
	R: ACCGCAGGACCCTACAGCAGTTACAT		
3	F: TTTCCGCTATTCCATTCCGAGCT	4546-6780	2235bp
	R: CCTGTACCAACTGAGCAAGTTCAATTCT		
4	F: AATCCTGCGGGATGAATCATGAGT	6583-9310	2728bp
	R: CTCTAAACAAGCACAGCTGTCCATGAG		
5	F: CCGAGTCTCCCTCCATGCCAAACT	9053-11513	2461bp
	R: AGGGGTCCAAATATACTGTGGAGTTGACA		
6	F: CGGTGTTGGAAATTGATGTTGGACT	11348-13852	2505bp
	R: GAGGGCGCCATAGGAGACGATATGT		
7	F: TCAGCGCCAACGGAAACAGCAGCT	13692-15231	1540bp
	R: AATTTCGGCCGTGTGGTTCCCGCCAAT		
8	F: ATGACGTATAGGTGTTGGCTCTATGCCA	1-2158	2158bp
	R: AGCCTAGCCAAGCATTCTTCAAGACTT		
9	F: TTGAGAGCTGCTGCTGCTCTCAGAAT	2066-4112	2146bp
	R: ATCCAATTGGGCATACGCGATGGGTT		
10	F: GCACCAAAGGGCATGGACCCTAT	4099-6497	2399bp
	R: ATGTTGGATGCGGAAACAAAGCACTT		
11	F: TTGAGGGAAGGGGTGTCGCAATCCT	6382-8833	2452bp
	R: AGATCATCCGAGTAGACAATCAGGGGCT		
12	F: ATGGTGCTCAGTTACTTCAAAAGCGGT	8744-11747	3044bp
	R: ATCAACAATGGAAACCAAGAACTCCGTG		
13	F: GAATCGGATACAGCGTATCTGTACGAGT	11511-1356	2057bp
	R: AGCCAATCTGTGCCATTCAGCTCACAT		
14	F: ATGTGCGGCTGCTTCACTTCATGACACCT	13302-15020	1719bp
	R: AATTTCGGCCGCATGGTTCTCGCC		

Supplementary Table S2

Information of reference strains

NO	Isolate	Country	year	Acession no
1	VR-2332	USA	1992	U87392
2	RespPRRS MLV	USA	1994	AF066183
3	Lelystad virus	Europe	1991	M96262
4	BJ-4	China	2000	AF331831
5	HB-1(sh)/2002	China	2002	AY150312
6	CH-1a	China	1996	AY032626
7	JXA1	China	2006	EF112445
8	HUN4	China	2007	EF635006.1
9	NADC30	USA	2012	JN654459
10	JL580	China	2015	KR706343
11	GD1404	China	2014	KT961415.1
12	QYYZ	China	2010	JQ308798
13	HNjz15	China	2015	KT945017
14	GM	China	2011	JN662414
15	QY1	China	2007	JN387271
16	JXwn06	China	2009	EF641008
17	JX/CH/2016	China	2016	KY495780

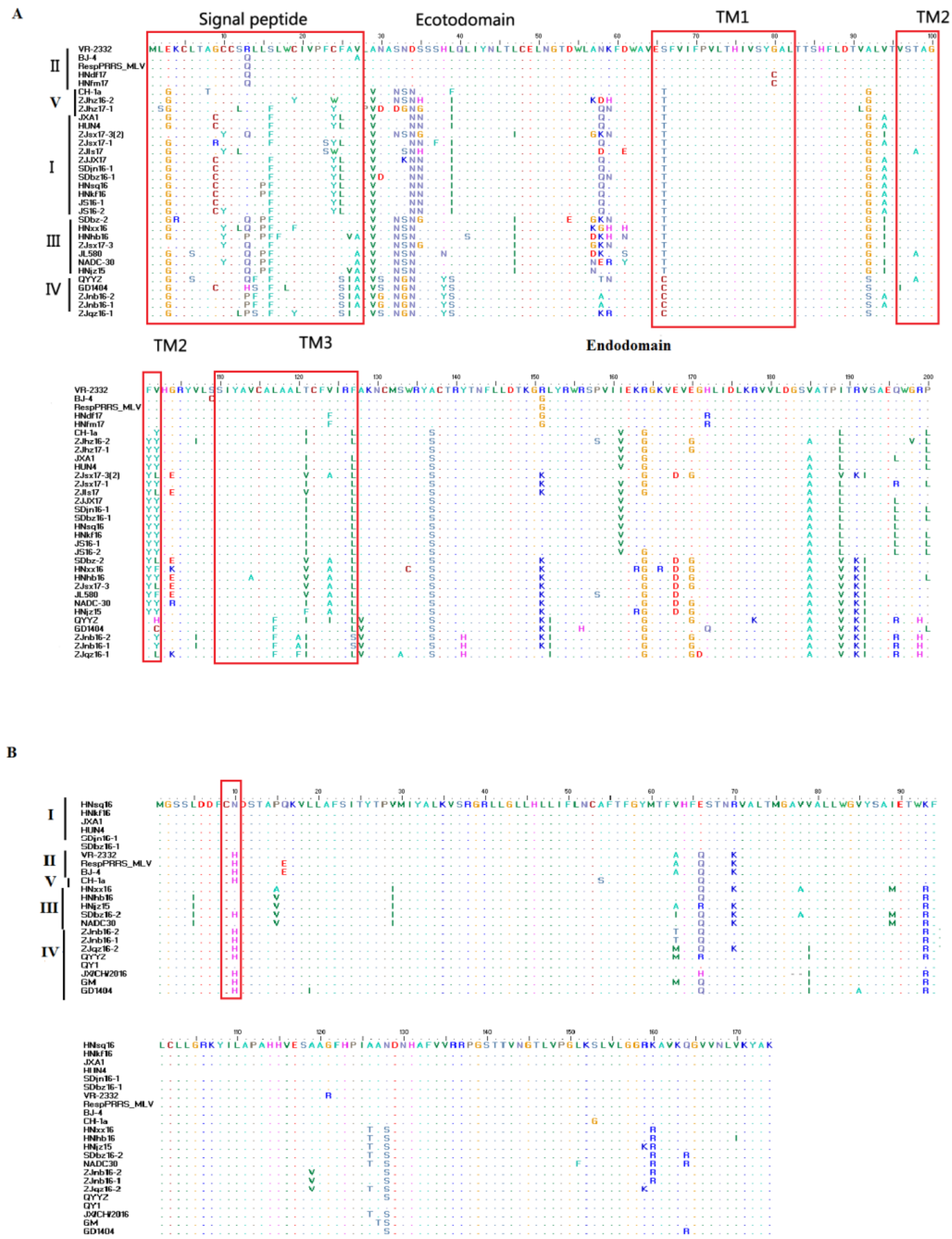

Supplmentary Figure 1 Analysis and comparison of amino acid mutations in GP5 (A), M (B). Regions of potential signal peptide, two hypervariable regions (HVR1 and HVR2), three transmembrane domains (TM1, TM2, and TM3) of GP5 and two key amino acids involved in cross neutralization reaction of M were marked with red box.

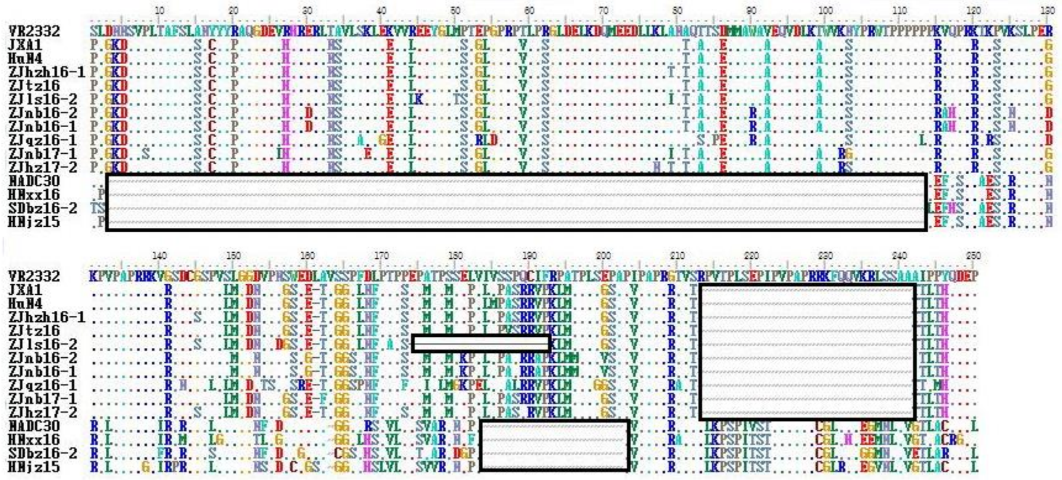

Supplemntary Figure 2 Multiple amino acid sequence comparison in NSP2.

Deletions in nsp2 coding region of different isolates were marked with black box.
